# Activating *PIK3CA* mutation promotes osteogenesis of bone marrow mesenchymal stem cells in macrodactyly

**DOI:** 10.1038/s41419-020-2723-6

**Published:** 2020-07-06

**Authors:** Hengqing Cui, Gang Han, Bin Sun, Xia Fang, Xinyi Dai, Shengbo Zhou, Hailei Mao, Bin Wang

**Affiliations:** 1 Department of Plastic and Reconstructive Surgery, Shanghai Ninth People’s Hospital, Shanghai JiaoTong University School of Medicine, Shanghai, China; 2 Department of Anesthesiology and Critical Care Medicine, Zhongshan Hospital, Fudan University, Shanghai, 200032 China

**Keywords:** Diseases, Cancer

## Abstract

Macrodactyly is a disabling congenital disease characterized by overgrowth of soft tissues and bones, which leads to finger enlargement and joint deformity. The mechanism of bone overgrowth in macrodactyly was rarely understood. In our study bone manifestations of three macrodactyly patients were analyzed by micro-CT. *PIK3CA* mutation was detected by next-generation sequencing (NGS) of a tumor gene-panel. The PI3K/AKT/mTOR pathway activation and target genes were analyzed. The osteogenic potential of macrodactyly-derived bone marrow mesenchymal stem cells (MAC-BMSCs) was compared with polydactyly-derived bone marrow mesenchymal stem cells (PD-BMSCs). *PIK3CA* inhibitors were tested for proliferation and osteogenesis potential of MAC-BMSCs. Activating *PIK3CA* mutations and activation of PI3K/AKT/mTOR pathway were detected in all MAC-BMSCs. MAC-BMSCs had enhanced osteogenesis potential compared with PD-BMSCs. *PIK3CA* knockdown by shRNA or BYL719 treatment significantly reduced osteogenic differentiation capacity of MAC-BMSCs. RNA-Seq and qRT-PCR revealed the upregulation of distal-less homeobox 5 (*DLX5*) in MAC-BMSCs compared with PD-BMSCs. The osteogenic potential of MAC-BMSCs was inhibited by *DLX5* knockdown, indicating that *DLX5* is a downstream target of *PIK3CA* activation-mediated osteogenesis. This study revealed that osteogenic differentiation in MAC-BMSCs is enhanced by *PIK3CA* activation mutation through PI3K/AKT/mTOR signaling pathway and can be reversed by *PIK3CA* knockdown or drug inhibition.

## Introduction

Macrodactyly is a rare congenital condition characterized by overgrowth of fingers or toes. It is a disabling disease, in which limb function and appearance are severely impaired. Overgrowth of bone, in addition to soft tissues, is often observed in macrodactyly and leads to enlarged finger size, abnormal bone formation and/or oblique fingers due to joint deformity. Oftentimes there is nerve involvement in the affected digits, among which median nerve is the most frequently involved^1^. Aberrant distribution of neurofilament has been reported in the affected nerve tissues^2^. Osseous enlargement, osteochondromatous proliferations, hypertrophic changes, and ankylosis of innervated joints can be observed in some areas of innervated bone tissue^3^. Mutation of *PIK3CA* can also be detected in the diseased nerve tissue^4^. To date, debulking surgery is the only effective intervention. However, although dubulking surgery of soft tissue enlargement could achieve satisfactory outcomes^5^, overgrowth of bone created greater challenge and often resulted in amputation in severe cases^6^. The pathogenesis of bone hyperplasia needs to be further studied. Bone marrow stem cells (BMSCs) are a type of precursor cells that have the potential to differentiate into osteoprogenitor cells and therefore are of great importance for bone homeostasis^7^. The destruction of osteogenic differentiation of BMSCs may lead to the imbalance of bone homeostasis^8^. Yang et al. found that macrodactyly-derived adipose mesenchymal stem cells have significantly increased capability of osteogenic differentiation^9^. However, the role of BMSCs in pathological conditions remains largely unknown.

In this study, we first detected *PIK3CA* somatic mutations in MAC-BMSCs. We investigated the role of PI3K/AKT/mTOR signaling pathway in osteogenic differentiation of MAC-BMSCs and explored the use of shRNA and p110α-specific inhibitor to block the osteogenic differentiation. Our study suggested *DLX5* as a potential biomarker for development of potential therapeutic treatment for macrodactyly.

## Methods

### Sample collection

Surgically amputated digits were collected from three patients with isolated macrodactyly and three patients with polydactyly in Department of Plastic and Reconstructive Surgery, Shanghai 9th People’s Hospital, followed by isolation of bone marrow mesenchymal stem cells (BMSCs). The detailed clinical information was listed in Table S1. This study was approved by the ethics committee of Shanghai 9th People’s Hospital (reference: 201580). Written informed consent of sample collection and photograph was obtained.

### DNA isolation and sequencing

Genomic DNA was extracted from bone marrow of amputated digits using QIAamp DNA Mini Kit (Qiagen, Valencia, CA, USA). DNA concentrations were measured with Qubit dsDNA HS assay kit (Life Technologies) on NanoDrop spectrophotometer (Thermo Fisher Scientific). Genomic DNA integrity was determined by agarose gel electrophoresis.

For targeted next-generation sequencing, genomic DNA was sheared to an average size of 200 bp using a Covaris S220 series sonicator. Fragmentation was followed by library construction with KAPA Hyper Prep kit (KAPA Biosystem, Roche) containing mixes for end repair, A‐tailing and adaptor ligation. Prepared library was hybridized for 16–24 h using custom capture DNA probes (Integrated DNA Technology Inc.). Target exons were captured by a panel designed by Shanghai Sinomics Corporation covering the coding exons of 593 genes which relate to clinical target therapy and pathogenetic mechanism of cancer (Table S2). The hybridized product was captured by streptavidin beads (Invitrogen) and amplified for 12 PCR cycles using KAPA HiFi HotStart ReadyMix. The amplified products were quantified by Qubit® 2.0 Fluorometer (Life Technologies, USA) and validated by Agilent 2100 bioanalyzer (Agilent Technologies, USA) to confirm the insert size and the mole concentration.

DNA sequencing was performed on HiSeq X-Ten sequencing system (Illumina) with 2 × 150 bp pair end sequencing.

Mutations detected in next generation were confirmed by Sanger sequencing. Primer sequences and additional details for polymerase chain reaction and Sanger sequencing are available upon request.

### Cell culture

BMSCs were isolated from amputated digits as described previously^10^. Briefly, bone marrow cells were washed out of the phalanx bones and centrifuged at 1000 × *g* for 5 min. Cells were cultured in HBMSCs medium (Cyagen, CA, USA) containing 10% fetal bovine serum, 1% penicillin and streptomycin, and maintained in a humidified atmosphere containing 5% CO_2_ at 37 °C. BMSCs were digested with 0.25% trypsin and passaged routinely when 80–90% confluence was reached. BMSCs from passages 3–6 were used in the following experiments.

### Cell proliferation assay

Both PD-BMSCs and MAC-BMSCs were seeded in 96-well plates at a density of 2000 cells per well. Cell proliferation assay was performed on days 1, 3, 5, and 7 using cell counting kit 8 (CCK-8, Dojindo, Japan), following the manufacture’s instruction. Briefly, 10 μL CCK-8 was added to each well and incubated for 2 h at 37 °C. The absorbance was measured at 562 nm using a microplate reader (Bio-Rad, Hercules, CA, USA). All experiments were repeated at least three times.

### Osteogenic differentiation

Osteogenic differentiation was conducted as previously described^11^. Briefly, MAC-BMSCs and PD-BMSCs were placed in a 12-well plate at a density of 5 × 10^4^ cells per well. Osteogenic differentiation was induced in basic growth medium supplemented with 100 nM dexamethasone (Cyagen, CA, USA) and 50 μM ascorbic acid (Cyagen, CA, USA). Osteogenic media was changed every 3 days. Quantitative real-time PCR examination of osteogenic marker genes were conducted 7 days after induction.

### Alkaline phosphatase (ALP) analysis and Alizarin red staining

ALP staining was performed 7 days after osteogenic induction according to the manufacturer’s instruction (Beyotime, Shanghai, China). Mineralized nodule formation was detected by 2% Alizarin red (Cyagen, CA, USA) 14 days after osteoblast differentiation. ALP activity of BMSCs was measured by a colorimetric enzymatic assay of alkaline phosphatase following the manufacturer’s protocol (Jiancheng, Nanjing, China). ALP activity was normalized to the total protein content determined by a bicinchoninic acid (BCA) kit (Thermo Fisher Scientific, USA). To quantify calcium accumulation, alizarin red was eluted by 10% cetylpyridinium chloride (CPC; Sigma) and absorbance at 570 nm was measured.

### Adipogenic differentiation

Adipogenic differentiation was conducted following the manufacturer’s instruction (Cyagen, CA, USA). Briefly, the MAC-BMSCs and PD-BMSCs were seeded in growth medium at a density of 5 × 10^4^ per well in a 12-well plate. The growth medium was changed every 3 days until the cells become fully confluent. The cells were incubated in adipogenic differentiation medium A (induction medium) for 3 days and the medium was replaced by adipogenic differentiation medium B (maintenance medium). After 24 h, the medium was changed back to adipogenic differentiation medium A. After the cycle of induction and maintenance was repeated for four times, cells were maintained in adipogenic differentiation medium B for additional 6 days until the lipid droplets are big and round enough. During this period, medium was changed every 3 days.

### Oil red O staining analysis

After the cells have differentiated, the adipogenic differentiation medium was removed. The cells were rinsed with phosphate-buffered saline (PBS) and fixed with 4% formaldehyde solution for 30 min. The cells were rinsed twice with 1× PBS and stained with 0.5 mL of oil red O working solution (3:2 dilution with distilled water and filter with filter paper) for 30 min. The wells were rinsed with 1× PBS for three times. Cells were visualized and analyzed under a microscope.

### RNA extraction and quantitative reverse transcription polymerase chain reaction (qRT-PCR)

Total RNA was extracted using the TRIzol reagent (Invitrogen, Carlsbad, CA, USA). The reverse transcription reaction was prepared on ice following the instructions of PrimeScript RT reagent Kit (TaKaRa, Shiga, Japan), and cDNA was obtained after the reaction completion. Quantitative RT-PCR was then performed by using Thunderbird SYBR qPCR Mix (Toyobo, Japan), with 10 μL of the total reaction system. The PCR amplification conditions were: predenaturation at 95 °C for 2 min, followed by 40 cycles of 95 °C for 20 s, 60 °C for 20 s, and 72 °C for 20 s. The primer sequences were listed in Table S3. The relative quantification of gene expression was analyzed with the values of 2^–ΔΔCT^, normalized with GAPDH expression level. Triplicate tests were conducted in each experiment.

### Western blot analysis

Cells were lysed by RIPA Lysis Buffer (Beyotime, China) supplemented with phosphatase inhibitor for total protein extraction. The concentration of each protein sample was quantified by a BCA (bicinchoninic acid) kit (Thermo Fisher Scientific, USA). Equal amounts of protein were separated by gel electrophoresis and then transferred to PVDF (polyvinylidene difluoride) membranes (Millipore, Billerica, MA, USA). After being blocked with 5% bovine serum albumin (BSA) in TBST for 30 min at room temperature, the PVDF membrance was incubated with primary antibodies (catalog numbers are listed in Table S3) at 4 °C overnight. After washing three times in TBST for 5 min/wash, the membranes were incubated with HRP-conjugated secondary antibody (Beyotime, A0208, 1:8000) for 45 min at room temperature. Protein bands were exposed via enhanced chemiluminescence (ECL, Thermo Fisher Scientific, Inc.) method.

### Knockdown of *PIK3CA* and *DLX5* expression by short hairpin RNA

MAC-BMSCs were seeded at 3 × 10^5^ cells per well into a 6-well plate and allowed to settle overnight. BMSCs were infected with lentivirus (MOI = 40) for 3 h in the presence of polybrene (8 μg/ml) and then maintained in regular medium. Forty-eight hours later, 4 μg/ml of puromycin was added and cells were cultured until all cells in the control group were dead. *PIK3CA* expression and PI3K/AKT/mTOR suppression were analyzed 3 days after puromycin selection.

### BYL719 administration

MAC-BMSCs were treated with BYL719 (Selleck, Houston, TX, USA) at the dose of 1.25, 2.5, or 5 μM 24 h after cell attachment. The protein expression of PI3K/AKT/mTOR pathway was detected by western blotting 2 h after treatment.

### RNA sequencing and data analysis

Total RNAs was extracted from passage 3 of cultured MAC-BMSCs (*n* = 2) and PD-BMSCs (*n* = 2) by using RNeasy mini kit (Qiagen, Germany). Complementary DNA library preparation and sequencing were performed according to Illumina standard protocol. Raw data of gene expression profiles of human bone marrow stem cells derived from macrodactyly and polydactyly patients, were submitted to the GEO database (https://www.ncbi.nlm.nih.gov/geo/) and the Geo accession number is GSE 147823. Gene Ontology (GO) analysis was performed with DAVID online tool. Top GO categories were selected according to the *p* values of expression changes. Eight differentially expressed genes were randomly selected and validated by qRT-PCR. The primers are listed in Table S4.

### Statistics analysis

Data were showed as mean ± standard deviations from at least three independent experiments. Differences between groups were analyzed using one-way analysis of variance (ANOVA) followed by Tukey test. Data were considered statistically significant when *p* value is <0.05. All statistical analysis was performed by Prism software (GraphPad Software, La Jolla, CA, USA).

## Results

### Bone overgrowth in macrodactyly patients

**Fig. 1:**
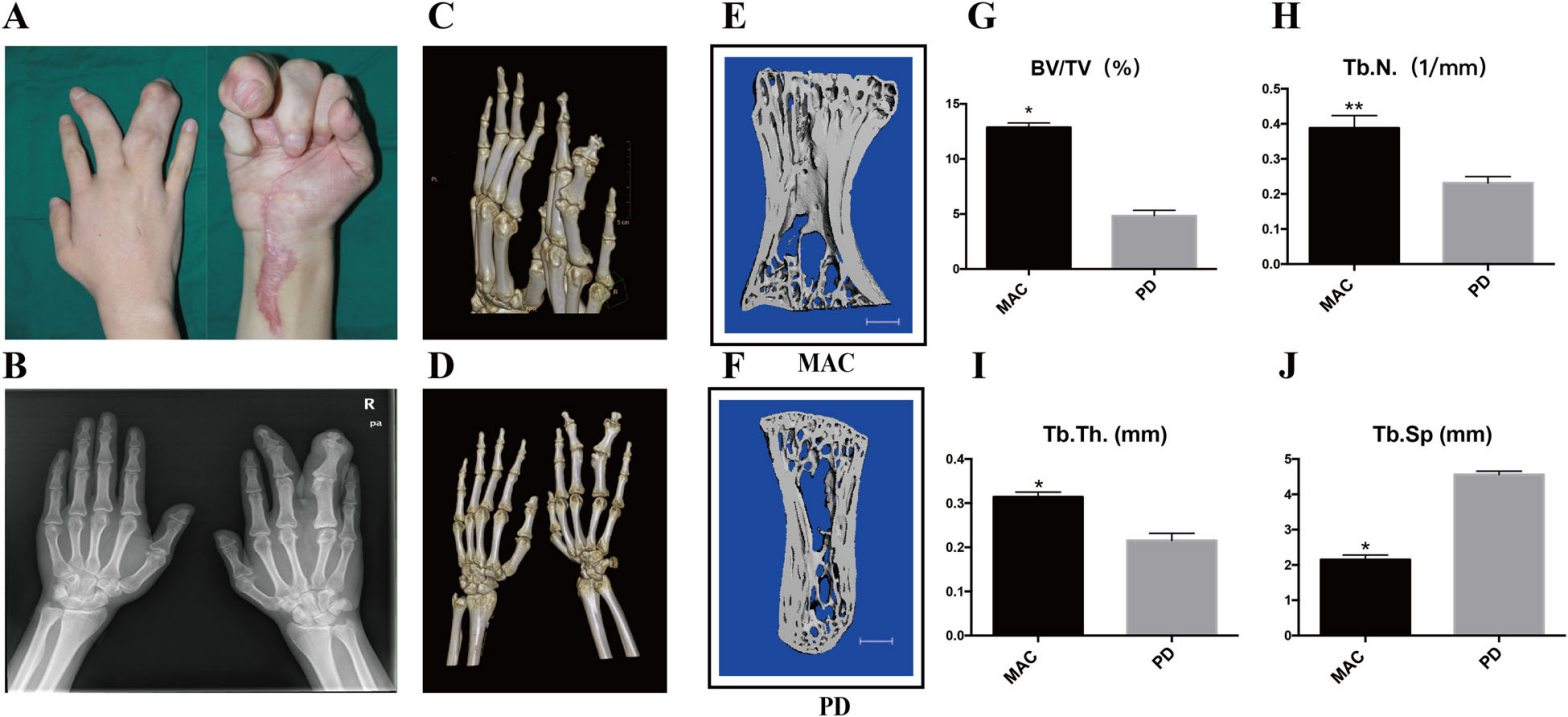
Clinical manifestation in patients with macrodactyly. **a** A macrodactyly patient with enlarged middle and ring fingers. **b** Radiograph demonstrating the hyperplastic bone formation compared with the normal hand. **c**, **d** Computed tomography (CT) with 3D reconstruction. **e**, **f** Micro-CT images of phalanxs from macrodactyly (MAC) and polydactyly (PD) patients (scale bar = 1.0 mm). **g**–**j** Quantitative parameters of Micro-CT. BV/TV: bone volume/total volume, Tb.N.: trabecular number, Tb.Th.: trabecular thickness, Tb.Sp: trabecular separation (**g**, **h**, **i**, **j** two-tailed unpaired *t*-test, *p < 0.05, **p < 0.01).

Radiographic examination of macrodactyly patient #1 showed enlarged bone volume as well as increased soft tissue volume in the right ring finger (Fig. 1a). The X-ray plain film revealed that the bone volume of the middle finger and ring finger of the right hand increased, and the bone mineral density was higher than that of the healthy fingers (Fig. 1b). CT three-dimensional reconstruction showed that there were joint deformities in the proximal joint of the middle and ring fingers as well as oblique finger deformities on the right hand (Fig. 1c, d). Micro-computed tomography (micro-CT) examination indicated an approximately twofold increase of bone volume fraction (BV/TV) in the macrodactylous phalanx compared with the phalanx of polydactyly (Fig. 1g). In addition, macrodactylous bones showed increased trabecular number (Tb.N., Fig. 1h), trabecular thickness (Tb.Th., Fig. 1i) and reduced trabecular separation (Tb.sp., Fig. 1j). These data demonstrated bone hyperplasia in macrodactyly.

### *PIK3CA*-mutated MAC-BMSCs displayed increased proliferation and osteogenesis potential

**Fig. 2:**
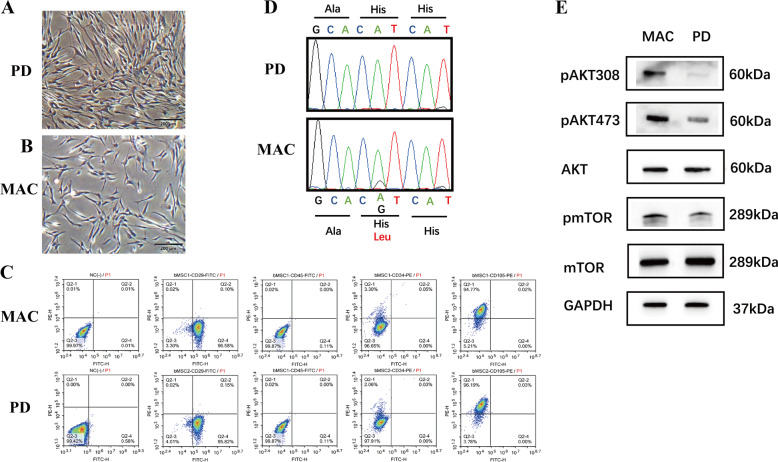
Similar cell morphology and surface marker expression of PD-BMSCs and Mac-BMSCs. **a**, **b** Cell morphology of PD-BMSCs (**a**) and MAC-BMSCs (**b**) (scale bar = 200 μm). **c** MAC-BMSCs (upper panel) and PD-BMSCs (lower panel) were stained with antibodies against BMSC-specific markers CD45, CD29, CD34, CD106, and analyzed by flow cytometry. **d** *PIK3CA* c.3140A>G (p.His1047Arg) mutation detected by Sanger sequencing in MAC-BMSCs but not in PD-BMSCs. **e** Protein expression of AKT/mTOR pathway was measured by western blotting.

To investigate whether BMSCs play a role in bone overgrowth in macrodactyly, we isolated BMSCs from amputated macrodactylous digits and compared with BMSCs from polydactyly patients. MAC-BMSCs (Fig. 2a) and PD-BMSCs (Fig. 2b) showed similar morphology in culture and expressed identical BMSC markers (Fig. 2c). We identified a somatic mutation of *PIK3CA* (c.3140A>G, p.H1047R) in MAC-BMSCs, but not in PD-BMSCs (Fig. 2d). Patients’ detailed information is listed in supplementary Table S1. Phosphorylation of AKT and mTOR was significantly increased in passage 3 MAC-BMSCs (Fig. 2e), indicating enhanced activity of the PI3K/AKT/mTOR signaling pathway.

**Fig. 3:**
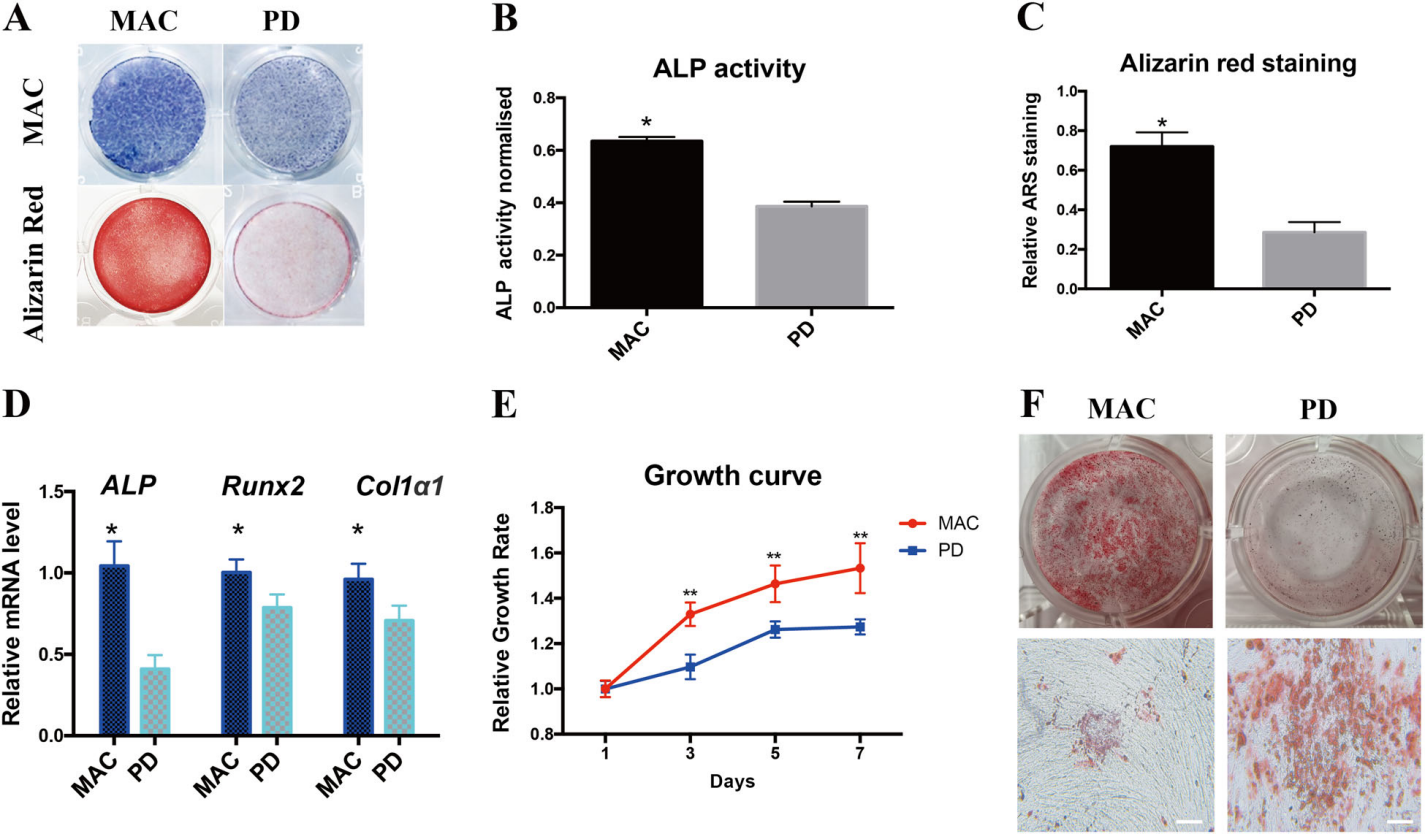
MAC-BMSCs showed higher osteoblast differentiation potential than PD-BMSCs. **a** ALP staining was performed on day 7 of osteogenic differentiation and alizarin red staining on day 14, respectively. **b** Quantitation of ALP activity on day 7 of differentiation. **c** Quantitation of Alizarin red staining on day 14 of differentiation. **d** Osteogenic differentiation was confirmed by qRT-PCR of marker genes on day 7. **e** Growth curve of MAC-BMSCs and PD-BMSCs. **f** Results of oil red staining for MAC-BMSCs and PD-BMSCs adipogenic differentiation (scale bar = 100 μm) (**b**–**e** two-tailed unpaired *t*-test, *p < 0.05, **p < 0.01).

Furthermore, MAC-BMSCs demonstrated higher osteoblast differentiation potential than PD-BMSCs when subjected to osteogenic induction, as indicated by increased Alkaline phosphatase (ALP) staining and Alizarin red staining (Fig. 3a). The quantitative analysis of the staining results revealed a significant difference between the two groups (Fig. 3b, c). Consistently, expression of osteoblast marker genes *collagen1α1* (*Col1α1*), *ALP*, and *Runx2* was increased in differentiated MAC-BMSCs (Fig. 3d). The cell growth curve showed that the proliferation rate of MAC-BMSCs was significantly higher than that of PD-BMSCs (Fig. 3e). Compared with wild-type cells, the adipogenic differentiation ability of *PIK3CA* mutant cells was significantly enhanced (Fig. 3f).

### Knockdown of *PIK3CA* reduced osteogenesis potential of MAC-BMSCs

**Fig. 4:**
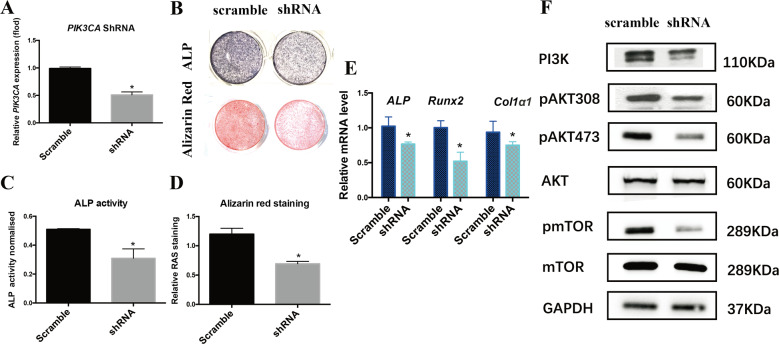
Downregulation of PIK3CA reduced osteogenic differentiation of MAC-BMSCs. **a** *PIK3CA* expression was determined by qRT-PCR in MAC-BMSCs transfected with shRNA targeting *PIK3CA* or scramble control. **b** ALP staining and Alizarin red staining were performed in MAC-BMSCs transfected with shRNA targeting *PIK3CA* or scramble control. **c**, **d** Intensity of ALP activity and Alizarin red staining was quantitated. **e** Osteogenic differentiation related marker genes including *ALP*, *Runx2*, and *Col1α1* were analyzed by qRT-PCR. **f** PI3K/AKT/mTOR pathway in *PIK3CA* knockdown MAC-BMSCs was analyzed by western blot (**a**, **c**, **d**, **e** two-tailed unpaired *t*-test, *p < 0.05, **p < 0.01).

PI3K/AKT/mTOR signaling pathway has been positively correlated with osteogenesis^12^. To investigate whether enhanced osteogenesis of MAC-BMSCs resulted from elevated PI3K signaling upon PIK3CA hyperactivation, we knocked down *PIK3CA* by lentivirus-mediated shRNA transfection in MAC-BMSCs. Decreased *PIK3CA* expression was confirmed by qRT-PCR (Fig. 4a) and western blot analysis (Fig. 4f). The intensity of ALP staining and Alizarin red staining in the *PIK3CA* knockdown group was much lower than the scramble RNA control group (Fig. 4b), and the quantitative analysis confirmed the statistical differences between the two groups (Fig. 4c, d). The osteogenic marker genes were also significantly downregulated in the knockdown group (Fig. 4e). These results showed that knocking down *PIK3CA* decreased the osteogenic potential of MAC-BMSCs, indicating an essential role of *PIK3CA* activity in regulating osteogenic differentiation of BMSCs in macrodactyly.

### Activated PI3K pathway enhanced osteogenic differentiation of PD-BMSCs

**Fig. 5:**
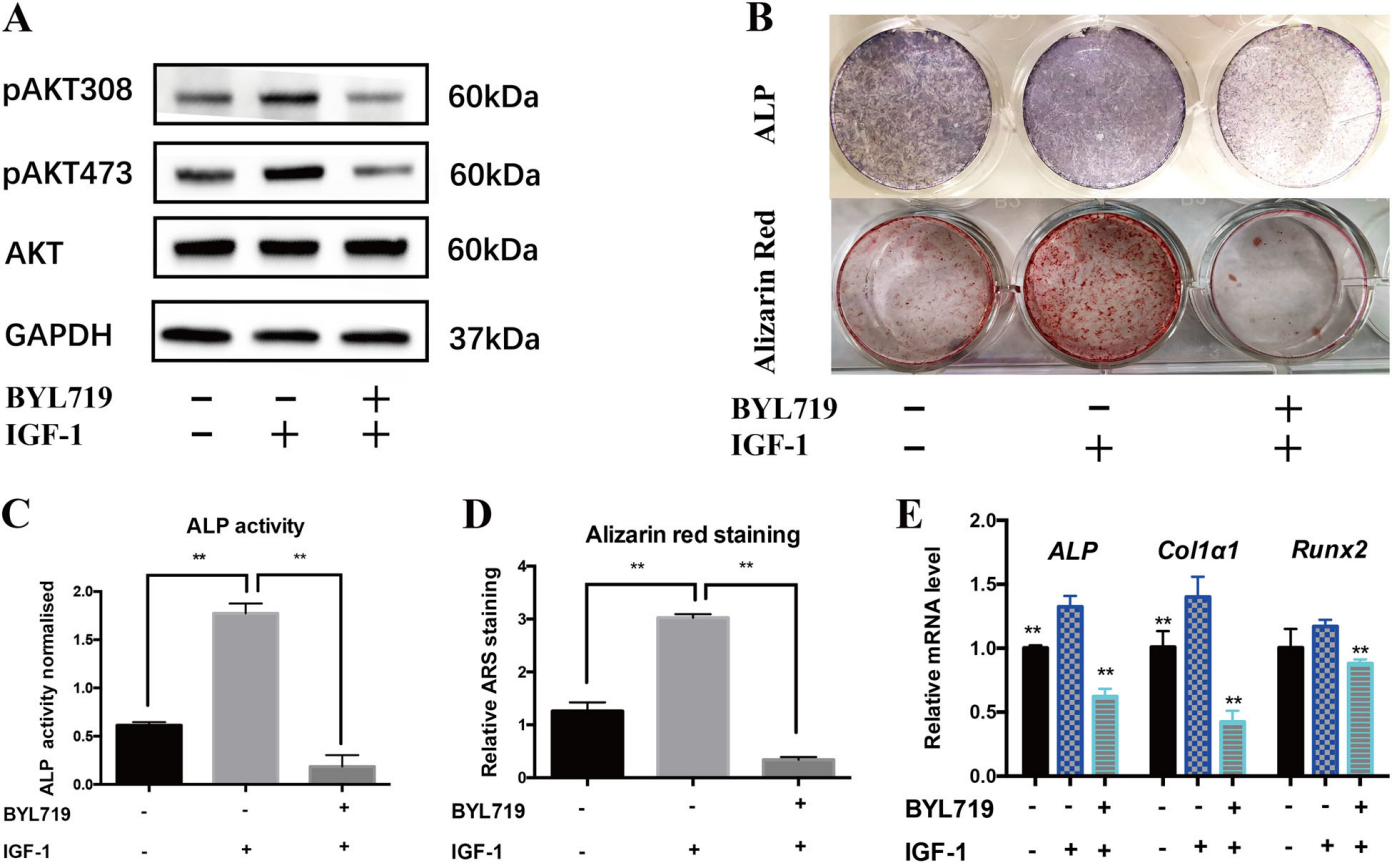
Activation of PI3K enhanced PD-BMSCs osteogenesis. **a** Protein levels of pAKT308, pAKT473, and total AKT were examined 2 h after treatment with IGF-1 and BYL719. **b** ALP staining and Alizarin red staining were performed and quantified in (**c**) and (**d**). **e** Relative mRNA expression levels of *ALP, Runx2*, and *Col1α1* were measured by qRT-PCR (**c**–**e** two-tailed unpaired *t*-test, *p < 0.05, **p < 0.01).

To further investigate if *PIK3CA* activation leads to elevated osteogenic potential of BMSCs, we activated PI3K signaling pathway in PD-BMSCs with IGF-1. Indeed, IGF-1 treatment promoted AKT phosphorylation (Fig. 5a) and osteogenesis of PD-BMSCs. Importantly, this effect can be greatly reversed by BYL719, an αspecific PI3K inhibitor. ALP and Alizarin red staining were significantly enhanced by IGF-1 in PD-BMSCs and the enhanced staining was significantly attenuated by BYL719 (Fig. 5b–d). Consistently, the expression of osteogenic marker genes *ALP, Runx2*, and *Col1α1* was increased by IGF-1 and inhibited by BYL719 (Fig. 5e), suggesting that the ability of osteogenesis induction by *PIK3CA* activation could be reversed by BYL719.

### BYL719 inhibited osteogenic potential of MAC-BMSCs

**Fig. 6:**
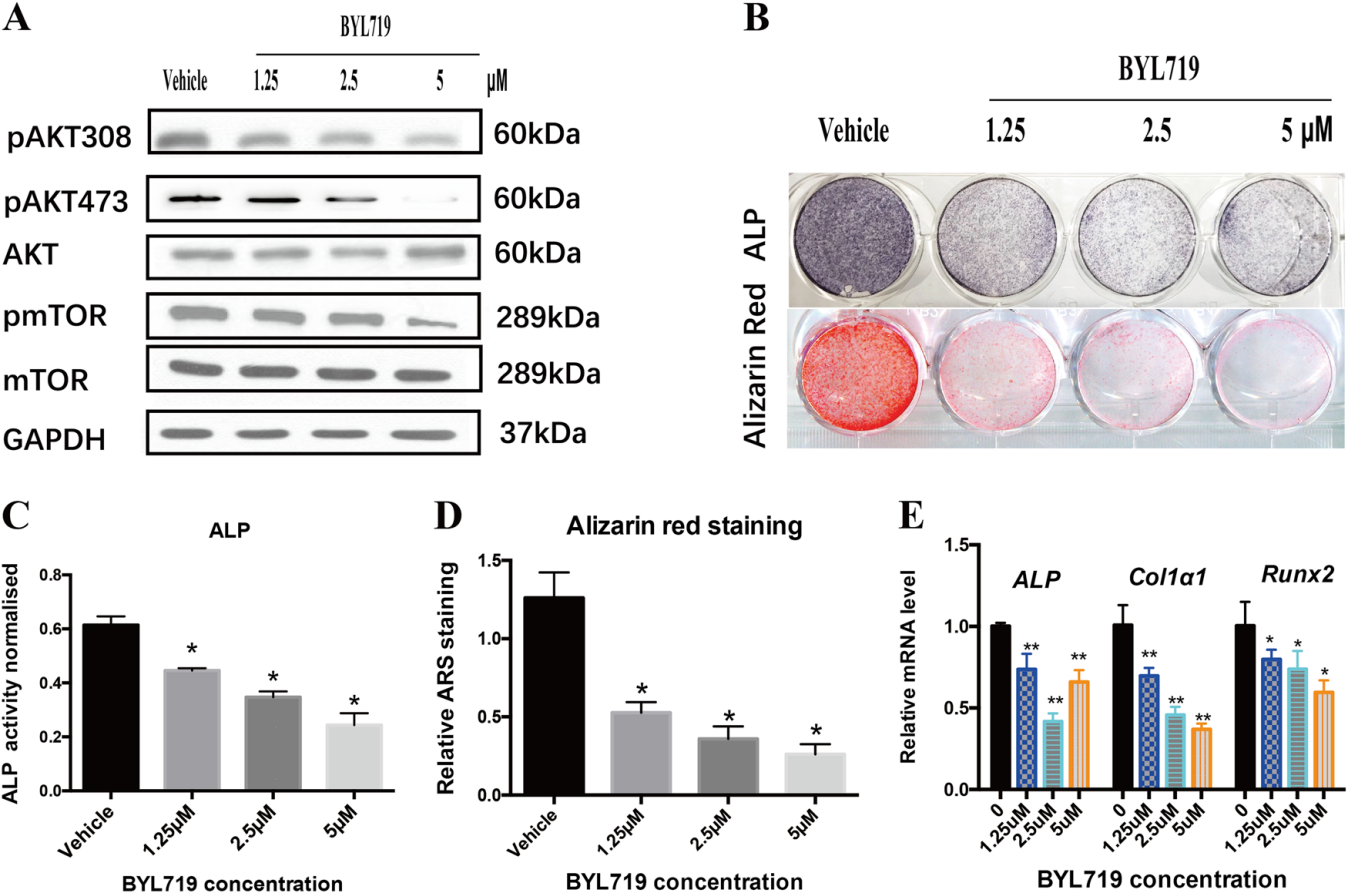
Suppression of MAC-BMSCs osteogenesis by BYL719. **a** Protein levels of pAKT308, pAKT473, total AKT, pmTOR, and total mTOR of MAC-BMSCs were examined 2 h after treatment with BYL719. **b** ALP staining and Alizarin red staining were performed and quantified in (**c**) and (**d**). **e** Relative mRNA expression levels of *ALP*, *Runx2*, and *Col1α1* were measured by qRT-PCR (**c**–**e** two-tailed unpaired *t*-test, *p < 0.05, **p < 0.01).

To look into the possibility of suppressing osteogenesis potential of MAC-BMSCs by pharmaceutical inhibition agent targeting at *PIK3CA*, we treated differentiating MAC-BMSCs with BYL719. BYL719 treatment inhibited phosphorylation of AKT and mTOR in a dose-dependent manner, suggesting effective inhibition of p110α activity (Fig. 6a). Consistently, BYL719 treatment resulted in decreased osteogenic differentiation of MAC-BMSCs in a dose-dependent manner, as shown by quantitative analysis of alkaline phosphatase staining (ALP) (Fig. 6c), alizarin red staining (Fig. 6d), and mRNA expression of *Col1α1, ALP,* and *Runx2* genes (Fig. 6e).

### *PIK3CA* regulated osteogenic differentiation via *DLX5*

**Fig. 7:**
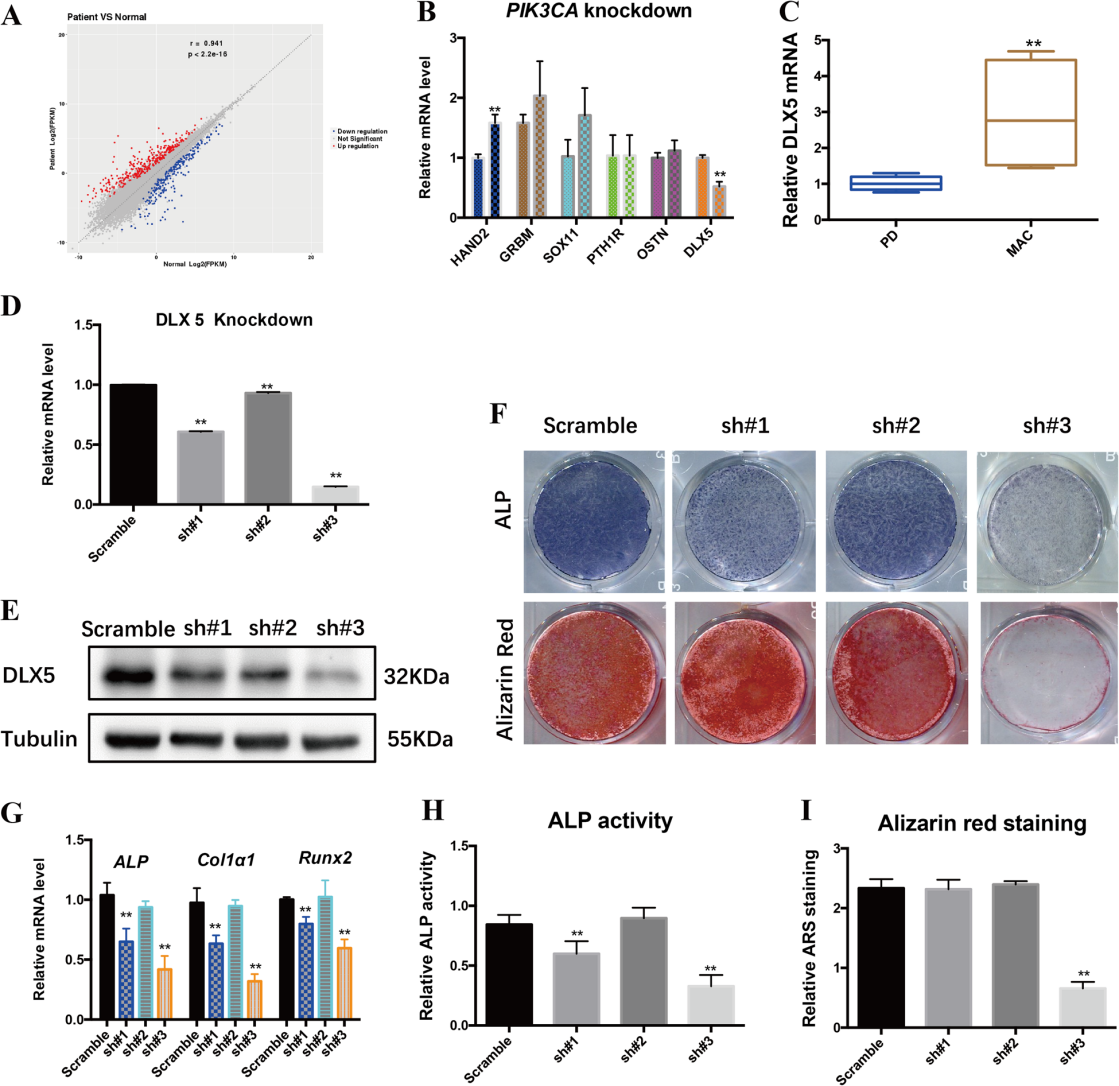
Osteogenesis of MAC-BMSCs by PIK3CA could be regulated through DLX5. **a** Scatterplot of differentially expressed genes (DEGs) between PD-BMSCs and MAC-BMSCs. **b** *DLX5* downregulated in *PIK3CA* knockdown group. **c** *DLX5* mRNA levels are higher in MAC-BMSCs compared with PD-BMSCs (*n* = 6). Data are plotted as whiskers (min to max). **d** *DLX5* mRNA expression level analyzed by qRT-PCR. **e** *DLX5* protein expression level analyzed by western blot. **f** ALP staining and alizarin red staining of *DLX5* KD MAC-BMSCs. **g** mRNA expression level of osteogenic marker genes. **h** Quantitative analysis of ALP activity. **i** Quantitative analysis of ARS (**c**–**i** two-tailed unpaired *t*-test, *p < 0.05, **p < 0.01).

To understand the mechanism of *PIK3CA*-mediated regulation of osteogenesis, we performed RNA-Seq analysis of PD-BMSCs and MAC-BMSCs. Differential expression analysis revealed that 383 genes were upregulated and 264 genes were downregulated in MAC-BMSCs versus PD-BMSCs (logFC < 1, *p* < 0.01, *p*-adj < 0.01) (Fig. 7a). Eight differentially expressed genes were randomly selected for validation by qRT-PCR as shown in Fig S1. Six of the 383 upregulated genes were associated with osteoblast differentiation. However, among the six genes, only *DLX5* was downregulated upon *PIK3CA* knockdown in MAC-BMSCs (Fig. 7b). The mRNA expression level of *DLX5* was also significantly higher in MAC-BMSCs (*n* = 6) than in PD-BMSCs (*n* = 6) (Fig. 7c). In the *DLX5* knockdown experiment, mRNA expression of *DLX5* was inhibited by the transfection of *DLX5* lentivirus shRNA (Fig. 7d), the expression level in Sh#3 was reduced to 16% of the control group. The protein expression of *DLX5* was significantly decreased at the same time (Fig. 7e). In the *DLX5* knockdown MAC-BMSCs, the osteogenic induction and differentiation ability of MAC-BMSCs was significantly decreased, as indicated by ALP staining and Alizarin red staining (Fig. 7f) and osteogenic marker gene expression analysis (Fig. 7g). The results of quantitative analysis also confirmed the above results (Fig. 7h, i). Collectively, these data suggest that *DLX5* regulation by *PIK3CA* could play an important role in osteogenesis.

## Discussion

Macrodactyly is one of the *PIK3CA* related overgrowth spectrum (PROS) caused by somatic mutation of *PIK3CA*^13^, which encodes the catalytic α-subunit of PI3K (p110α). PI3K catalyzes the conversion of phosphatidylinositol-4,5-bisphosphate (PIP_2_) to phosphatidylinositol (3,4,5)-triphosphate (PIP_3_)^14^. PIP_3_ recruits AKT and promotes PDK1 phosphorylation of AKT at Thr308^15^. H1047R, E542K, and C420R mutations are located in the kinase domain, helical domain, and C2 domain, respectively. H1047R and E542K are ‘hot-spot’ mutations, and C420R is frequently detected in macrodactyly. Many studies have shown that overexpression of *PIK3CA* with H1047R, E542K, and C420R point mutations in different cell types can induce a gain-of-function activation PI3K-AKT-mTOR pathway and cause phenotypic changes such as enhanced proliferation and invasion^16–19^. We also demonstrated this in bone marrow mesenchymal stem cells derived from macrodactyly in our research. Although PI3K/AKT/mTOR cell-signaling pathway has been shown to play an important role in macrodactyly^13,20,21^, whether or not this pathway contributes to bone malformation in macrodactyly was unknown.

Overgrowth of bone in macrodactyly is a great challenge for hand surgeons.

In this study, we demonstrated that *PIK3CA* was mutated in BMSCs derived from overgrown bones in macrodactyly patients and the activating mutations resulted in hyperactivation of PI3K/AKT/mTOR signaling pathway and enhanced osteogenic differentiation. We further explored the downstream osteogenesis pathway and identified *DLX5* gene as a potential target gene. Bone overgrowth is a common feature in macrodactyly patients. Bone formation relies on BMSCs which give rise to new osteoblasts and their progenitors^22^. PI3K/AKT pathway has been reported to promote osteoblast differentiation and accumulation of bone^23–25^. We showed that *PIK3CA* gene was mutated in MAC-BMSCs which resulted in hyperactivation of PI3K/AKT/mTOR pathway while no *PIK3CA* mutation was detected in PD-BMSCs. Importantly, MAC-BMSCs displayed higher osteogenic potential than PD-BMSCs. Consistent with the role of PI3K signaling in osteogenesis, MAC-BMSCs exhibited higher osteogenic potential than PD-BMSCs. We further demonstrated that knocking-down of PIK3CA in MAC-BMSCs led to inhibited osteogenesis while activation of PI3K signaling in PD-BMSCs resulted in enhanced osteogenesis. To our knowledge, this is the first study revealing the molecular mechanism of bone overgrowth in macrodactyly.

By comparing the transcriptome profiling of MAC-BMSCs versus PD-BMSCs, we identified *DLX5* as a differentially expressed osteogenesis-related gene and the only gene that responded to *PIK3CA* knockdown among a series of osteogenic genes. *DLX5* can bind to *Runx2* enhancer and drive *Runx2* expression, which is a key regulator of BMSC osteogenic differentiation^26^. Importantly, Dai et al. demonstrated that mTORC1-S6K1 regulates osteogenesis via *DLX5* regulation of *Runx2*^27^. Therefore, *DLX5* may serve as a downstream effector of PI3K-mediated hyper-osteogenesis of MAC-BMSCs.

Understanding the involvement of *PIK3CA* hyperactivation in osteogenesis of MAC-BMSCs provides an opportunity to pharmaceutically inhibit the bone overgrowth in macrodactyly. BYL719 is a selective inhibitor for p110α which has been approved to treat *PIK3CA*-mutated breast cancer. Venot et al. administered BYL719 to 19 patients with PROS disorders, showing dramatic clinical improvement in suppressing tissue overgrowth and even in correcting the skeletal deformity^21^. Notably, recipients in the study displayed good toleration. Only three patients had herglycaemia and no other side effects were observed. This study suggests a therapeutic strategy to use BYL719 as a potential drug to prevent abnormal bone formation in macrodactyly. The impact of *PIK3CA* blockage on cell survival is inevitable^28^. The decrease in staining due to inhibition of cell proliferation was corrected by normalizing the quantity of alkaline phosphatase staining to total protein. The expression of osteogenic marker genes (*alp, runx2*, and *col1α1*) was evaluated to confirm the inhibitory effect of BYL719 on MAC-BMSCs osteogenesis. Our study demonstrated that the *PIK3CA*-specific small molecule inhibitor can effectively reduce osteogenesis of MAC-BMSCs, showing a promising therapeutic potential.

In summary, we reported for the first time the possible mechanism of hyperplastic bone formation in macrodactyly. We identified *PIK3CA* mutations in BMSCs from macrodactylous bones and demonstrated the role of PI3K/AKT activation in the osteogenesis of MAC-BMSCs. Our findings provide the theoretical basis for targeted therapy of macrodactyly by inhibiting key regulators such as PI3K and DLX5.

## Supplementary information

table S4

table S3

table S2

table S1

Supplementary Figure Legends
