# Supplementary material for: Activating PIK3CA mutation promotes osteogenesis of bone marrow mesenchymal stem cells in macrodactyly
Source: Cell Death Dis. 2020 Jul 6;11(7):505. doi: 10.1038/s41419-020-2723-6 (PMC7338441; doi:10.1038/s41419-020-2723-6)
Supplement: Supplementary file 1 — table S4 [file 41419_2020_2723_MOESM1_ESM.docx]

| Gene name | Sense | Antisense |
| --- | --- | --- |
| *ALP* | ACGAGCTGAACAGGAACAACGT | CACCAGCAAGAAGAAGCCTTTG |
| *Runx2* | ACAGTAGAT GGACCTCGGGA | ATACTGGGATGAGGAATGCG |
| *Col1α1* | AGGGCCAAGACGAAGACATC | AGATCACGTCATCGCACAAC |
| *GAPDH* | TGGTATCGTGGAAGGACTCATGAC | ATGCCAGTGAGCTTCCCGTTCAGC |
| DLX5 | TACCCAGCCAAAGCTTATGCCG | GCCATTCACCATTCTCACCTCG |
| HAND2 | GGCAGAGATCAAGAAGACCGAC | CGGCCTTTGGTTTTCTTGTCGTT |
| GRBM | TCATCAACCGCTTCTGTTACGGC | CAGAAGGAGCAGGACTGAAAGG |
| SOX11 | GCTGAAGGACAGCGAGAAGATC | GGGTCCATTTTGGGCTTTTTCCG |
| PTH1R | TCACCGTAGCTGTGCTCATCCT | GAGTAGAGCACAGCGTCCTTGA |
| OSTN | CAGGAAAAGTCCTCTCAGTAGATG | GCAAGAGTTTTGCTGTCAGGTCA |
