# Supplementary material for: Activating PIK3CA mutation promotes osteogenesis of bone marrow mesenchymal stem cells in macrodactyly
Source: Cell Death Dis. 2020 Jul 6;11(7):505. doi: 10.1038/s41419-020-2723-6 (PMC7338441; doi:10.1038/s41419-020-2723-6)
Supplement: Supplementary file 2 — table S3 [file 41419_2020_2723_MOESM2_ESM.docx]

| Primary antibodies | Dilution | Supplier&Cat.no |
| --- | --- | --- |
| mTOR Rabbit mAb | 1:5000 TBST 5%BSA | CST #2983S |
| Phospho-mTOR (Ser2448) Rabbit mAb | 1:5000 TBST 5%BSA | CST #5536T |
| Akt (pan) Rabbit mAb | 1:2000 TBST 5%BSA | CST #4691T |
| Phospho-Akt (Ser473) Rabbit mAb | 1:2000 TBST 5%BSA | CST #4060S |
| Phospho-Akt (Thr308) Rabbit mAb | 1:1000 TBST 5%BSA | CST #13038T |
| PI3 Kinase p110α Rabbit mAb | 1:1000 TBST 5% milk | CST #4249S |
| Anti-GAPDH antibody | 1:10000 TBST 5% milk | Abcam ab181602 |
