# Supplementary material for: Activating PIK3CA mutation promotes osteogenesis of bone marrow mesenchymal stem cells in macrodactyly
Source: Cell Death Dis. 2020 Jul 6;11(7):505. doi: 10.1038/s41419-020-2723-6 (PMC7338441; doi:10.1038/s41419-020-2723-6)
Supplement: Supplementary file 3 — table S2 [file 41419_2020_2723_MOESM3_ESM.docx]

| ABL1 | ABL2 | ABRAXAS1 | ACVR1 | ACVR1B | ADGRA2 | AGO2 | AKT1 | AKT2 | AKT3 |
| --- | --- | --- | --- | --- | --- | --- | --- | --- | --- |
| ALK | ALOX12B | AMER1 | ANKRD11 | APC | AR | ARAF | ARFRP1 | ARID1A | ARID1B |
| ARID2 | ARID5B | ASXL1 | ASXL2 | ATM | ATR | ATRX | AURKA | AURKB | AXIN1 |
| AXIN2 | AXL | B2M | BABAM1 | BAP1 | BARD1 | BBC3 | BCL10 | BCL2 | BCL2L1 |
| BCL2L11 | BCL2L2 | BCL6 | BCOR | BCORL1 | BIRC3 | BLM | BMPR1A | BRAF | BRCA1 |
| BRCA2 | BRD4 | BRIP1 | BTG1 | BTG2 | BTK | CALR | CARD11 | CARM1 | CASP8 |
| CBFB | CBL | CCND1 | CCND2 | CCND3 | CCNE1 | CD22 | CD274 | CD276 | CD70 |
| CD79A | CD79B | CDC42 | CDC73 | CDH1 | CDK12 | CDK4 | CDK6 | CDK8 | CDKN1A |
| CDKN1B | CDKN2A | CDKN2B | CDKN2C | CEBPA | CENPA | CHD2 | CHD4 | CHEK1 | CHEK2 |
| CIC | CREBBP | CRKL | CRLF2 | CSDE1 | CSF1R | CSF3R | CTCF | CTLA4 | CTNNA1 |
| CTNNB1 | CUL3 | CUL4A | CXCR4 | CYLD | CYP17A1 | CYSLTR2 | DAXX | DCUN1D1 | DDR1 |
| DDR2 | DICER1 | DIS3 | DNAJB1 | DNMT1 | DNMT3A | DNMT3B | DOT1L | DROSHA | DUSP4 |
| E2F3 | EED | EGFL7 | EGFR | EIF1AX | EIF4A2 | EIF4E | ELF3 | ELOC | EMSY |
| EP300 | EPAS1 | EPCAM | EPHA3 | EPHA5 | EPHA7 | EPHB1 | EPHB4 | ERBB2 | ERBB3 |
| ERBB4 | ERCC2 | ERCC3 | ERCC4 | ERCC5 | ERF | ERG | ERRFI1 | ESR1 | ETV1 |
| ETV6 | EZH1 | EZH2 | FAM46C | FAM58A | FANCA | FANCC | FANCD2 | FANCE | FANCF |
| FANCG | FANCL | FAS | FAT1 | FBXW7 | FGF10 | FGF12 | FGF14 | FGF19 | FGF23 |
| FGF3 | FGF4 | FGF6 | FGFR1 | FGFR2 | FGFR3 | FGFR4 | FH | FLCN | FLT1 |
| FLT3 | FLT4 | FOXA1 | FOXL2 | FOXO1 | FOXP1 | FRS2 | FUBP1 | FYN | GABRA6 |
| GATA1 | GATA2 | GATA3 | GATA4 | GATA6 | GID4 | GLI1 | GNA11 | GNA13 | GNAQ |
| GNAS | GPS2 | GREM1 | GRIN2A | GRM3 | GSK3B | H3F3A | H3F3B | H3F3C | HDAC1 |
| HGF | HIST1H1C | HIST1H2BD | HIST1H3A | HIST1H3B | HIST1H3C | HIST1H3D | HIST1H3E | HIST1H3F | HIST1H3G |
| HIST1H3H | HIST1H3I | HIST1H3J | HIST2H3C | HIST2H3D | HIST3H3 | HLA-A | HLA-B | HNF1A | HOXB13 |
| HRAS | HSD3B1 | HSP90AA1 | ICOSLG | ID3 | IDH1 | IDH2 | IFNGR1 | IGF1 | IGF1R |
| IGF2 | IKBKE | IKZF1 | IL10 | IL7R | INHA | INHBA | INPP4A | INPP4B | INPPL1 |
| INSR | IRF2 | IRF4 | IRS1 | IRS2 | JAK1 | JAK2 | JAK3 | JUN | KAT6A |
| KDM5A | KDM5C | KDM6A | KDR | KEAP1 | KEL | KIT | KLF4 | KLHL6 | KMT2A |
| KMT2B | KMT2C | KMT2D | KMT5A | KNSTRN | KRAS | LATS1 | LATS2 | LMO1 | LRP1B |
| LTK | LYN | LZTR1 | MAF | MAGI2 | MALT1 | MAP2K1 | MAP2K2 | MAP2K4 | MAP3K1 |
| MAP3K13 | MAP3K14 | MAPK1 | MAPK3 | MAPKAP1 | MAX | MCL1 | MDC1 | MDM2 | MDM4 |
| MED12 | MEF2B | MEN1 | MERTK | MET | MGA | MITF | MKNK1 | MLH1 | MPL |
| MRE11 | MSH2 | MSH3 | MSH6 | MSI1 | MSI2 | MST1 | MST1R | MTAP | MTOR |
| MUTYH | MYC | MYCL | MYCN | MYD88 | MYOD1 | NBN | NCOA3 | NCOR1 | NEGR1 |
| NF1 | NF2 | NFE2L2 | NFKBIA | NKX2-1 | NKX3-1 | NOTCH1 | NOTCH2 | NOTCH3 | NOTCH4 |
| NPM1 | NRAS | NSD1 | NSD2 | NSD3 | NT5C2 | NTHL1 | NTRK1 | NTRK2 | NTRK3 |
| NUF2 | NUP93 | P2RY8 | PAK1 | PAK3 | PAK5 | PALB2 | PARP1 | PARP2 | PARP3 |
| PAX5 | PBRM1 | PDCD1 | PDCD1LG2 | PDGFRA | PDGFRB | PDK1 | PDPK1 | PGR | PHOX2B |
| PIK3C2B | PIK3C2G | PIK3C3 | PIK3CA | PIK3CB | PIK3CD | PIK3CG | PIK3R1 | PIK3R2 | PIK3R3 |
| PIM1 | PLCG2 | PLK2 | PMAIP1 | PMS1 | PMS2 | PNRC1 | POLD1 | POLE | PPARG |
| PPM1D | PPP2R1A | PPP2R2A | PPP4R2 | PPP6C | PRDM1 | PRDM14 | PREX2 | PRKAR1A | PRKCI |
| PRKD1 | PRKDC | PRKN | PRSS8 | PTCH1 | PTEN | PTP4A1 | PTPN11 | PTPRD | PTPRO |
| PTPRS | PTPRT | QKI | RAB35 | RAC1 | RAC2 | RAD21 | RAD50 | RAD51 | RAD51B |
| RAD51C | RAD51D | RAD52 | RAD54L | RAF1 | RANBP2 | RARA | RASA1 | RB1 | RBM10 |
| RECQL | RECQL4 | REL | RET | RFWD2 | RHEB | RHOA | RICTOR | RIT1 | RNF43 |
| ROS1 | RPS6KA4 | RPS6KB2 | RPTOR | RRAGC | RRAS | RRAS2 | RTEL1 | RUNX1 | RUNX1T1 |
| RXRA | RYBP | SDHA | SDHAF2 | SDHB | SDHC | SDHD | SESN1 | SESN2 | SESN3 |
| SETD2 | SF3B1 | SGK1 | SH2B3 | SH2D1A | SHOC2 | SHQ1 | SLIT2 | SLX4 | SMAD2 |
| SMAD3 | SMAD4 | SMARCA4 | SMARCB1 | SMARCD1 | SMO | SMYD3 | SNCAIP | SOCS1 | SOS1 |
| SOX10 | SOX17 | SOX2 | SOX9 | SPEN | SPOP | SPRED1 | SPTA1 | SRC | SRSF2 |
| STAG2 | STAT3 | STAT4 | STAT5A | STAT5B | STK11 | STK19 | STK40 | SUFU | SUZ12 |
| SYK | TAF1 | TAP1 | TAP2 | TBX3 | TCF3 | TCF7L2 | TEK | TERC | TERT |
| TET1 | TET2 | TGFBR1 | TGFBR2 | TIPARP | TMEM127 | TMPRSS2 | TNFAIP3 | TNFRSF14 | TOP1 |
| TOP2A | TP53 | TP53BP1 | TP63 | TRAF2 | TRAF7 | TSC1 | TSC2 | TSHR | TYRO3 |
| U2AF1 | UPF1 | VEGFA | VHL | VTCN1 | WISP3 | WT1 | WWTR1 | XIAP | XPO1 |
| XRCC2 | YAP1 | YES1 | ZBTB2 | ZFHX3 | ZNF217 | ZNF703 | BCR | CD74 | ETV4 |
| ETV5 | EWSR1 | EZR | MYB | NUTM1 | PDGFB | PRKACA | RSPO2 | SDC4 | SLC34A2 |
| HLA-C | HLA-DRB1 | HLA-DRB5 | HLA-DPB1 | HLA-DQB1 | HLA-DQA1 | HLA-DPA1 | ABCB1 | C8orf34 | CDA |
| CYP19A1 | CYP1B1 | CYP2C8 | CYP2D6 | DPYD | ERCC1 | GSTP1 | MTHFR | MTRR | NQO1 |
| NUDT15 | RRM1 | SEMA3C | SLC19A1 | SLC28A1 | SLC28A3 | SOD2 | TPMT | TYMS | UGT1A1 |
| WNT5B | XPC | XRCC1 |  |  |  |  |  |  |  |
