# Supplementary material for: Activating PIK3CA mutation promotes osteogenesis of bone marrow mesenchymal stem cells in macrodactyly
Source: Cell Death Dis. 2020 Jul 6;11(7):505. doi: 10.1038/s41419-020-2723-6 (PMC7338441; doi:10.1038/s41419-020-2723-6)
Supplement: Supplementary file 4 — table S1 [file 41419_2020_2723_MOESM4_ESM.docx]

| Patient ID | Sex | Age | Clinical Diagnosis | Affected Hand | PIK3CA mutation | Variant allele frequency % |
| --- | --- | --- | --- | --- | --- | --- |
| 1 | male | 25 years | macrodactyly | Right hand | H1047R | 18.6 |
| 2 | female | 1 years | macrodactyly | Left feet | E542K | 19.15 |
| 3 | female | 1 years | macrodactyly | Right hand | C420R | 26.81 |
| 4 | male | 1 years | polydactyly | Left hand | No | —— |
| 5 | male | 1 years | polydactyly | Right hand | No | —— |
| 6 | female | 17 years | polydactyly | Right hand | No | —— |
