# Supplementary material for: Activating PIK3CA mutation promotes osteogenesis of bone marrow mesenchymal stem cells in macrodactyly
Source: Cell Death Dis. 2020 Jul 6;11(7):505. doi: 10.1038/s41419-020-2723-6 (PMC7338441; doi:10.1038/s41419-020-2723-6)
Supplement: Supplementary file 5 — Supplementary Figure Legends [file 41419_2020_2723_MOESM5_ESM.docx]

**Supplymentary figure legends**

**Figure S1. Validation of RNA-Seq results by qRT-PCR.** Expressions of eight radomly selected differentially expressed genes revealed by RNA-Seq in two polydactyly patients and two macrodactyly patients were validated by qRT-PCR. The results of the two methods are consistent.

Table S1

The detailed clinical information of polydactyly and macrodactyly patients

Table S2

593 genes related to clinical target therapy and pathogenetic mechanism of cancer

Table S3

Primary antibodies list

Table S4

Primers for RT-qPCR
